# US healthcare costs attributable to type A and type B influenza

**DOI:** 10.1080/21645515.2017.1345400

**Published:** 2017-07-12

**Authors:** Songkai Yan, Derek Weycker, Stefania Sokolowski

**Affiliations:** a GSK, Philadelphia, PA, USA; b Policy Analysis Inc. (PAI), Brookline, MA, USA

**Keywords:** burden, healthcare costs, influenza, influenza A virus, influenza B virus, vaccine

## Abstract

While the overall healthcare burden of seasonal influenza in the United States (US) has been well characterized, the proportion of influenza burden attributable to type A and type B illness warrants further elucidation. The aim of this study was to estimate numbers of healthcare encounters and healthcare costs attributable to influenza viral strains A and B in the US during the 2001/2002 – 2008/2009 seasons. Healthcare encounters and costs in the US during the 2001/2002 – 2008/2009 seasons for influenza type A and influenza type B were estimated separately and collectively, by season and age group, based on data from published literature and secondary sources for: rates of influenza-related encounters requiring formal healthcare, unit costs of influenza-related healthcare encounters, and estimates of population size. Across 8 seasons, projected annual numbers of influenza-related healthcare encounters ranged from 11.3–25.6 million, and healthcare costs, from $2.0–$5.8 billion. While the majority of influenza illness was attributable to type A strains, type B strains accounted for 37% of healthcare costs across all seasons, and as much as 66% in a single season. The outpatient burden of type B disease was considerable among persons aged 18–64 y while the hospital cost burden was highest in young children. Influenza viral strain B was associated with considerable health system burden each year during the period of interest. Increasing influenza vaccine coverage, especially with the recently approved quadrivalent products including an additional type B strain, could potentially reduce overall annual influenza burden in the US.

## Introduction

Influenza immunization is currently the primary intervention to prevent influenza-related illness.^1^ Because numerous strains of influenza virus circulate at any time, and the proportion of prevalent strains changes over time, extensive global surveillance-based monitoring is undertaken to identify the specific combination of strains to target for inclusion in the influenza vaccine for the following season.^2^

Historically, recommendations for yearly vaccine viral targets have included 2 sub-strains of type A influenza, A (H1N1) and A (H3N2), and one sub-strain of type B influenza, B/Yamagata or B/Victoria. Resultant vaccines are “trivalent,” targeting 3 viral strains. Although the yearly recommendation of viral targets is based on rigorous surveillance efforts, a mismatch can occur between vaccine targets and prevalent influenza viruses encountered by humans during a given influenza season.^3^ Both lineages of influenza virus are known to co-circulate and it is reported that vaccine target to prevalent B viral mismatch occurred in approximately 50% of recent influenza seasons.^4^

Accordingly, despite systematic surveillance efforts, the availability of effective vaccines and widespread vaccine coverage, seasonal influenza continues to be responsible for substantial morbidity and mortality in the United States (US): 610,660 lost life years, 3.1 million hospital days, 31.4 million outpatient visits, $10.4 billion in healthcare costs, and $16.3 billion in morbidity/mortality-related lost earnings.^5^ It is largely unknown, however, the extent to which the burden of influenza is attributable to type A illness versus type B illness. The limited evidence that does exist suggests that when influenza type B activity is heightened, the impact on mortality and hospitalizations is comparable to that of influenza type A, and that the clinical and cost burden of influenza type B has increased in recent years.^6,7,8^ A better understanding of the population-level annual burden of viral type A and B influenza illness in the US healthcare system is needed.

## Results

### Base case analyses

**Figure 1.**
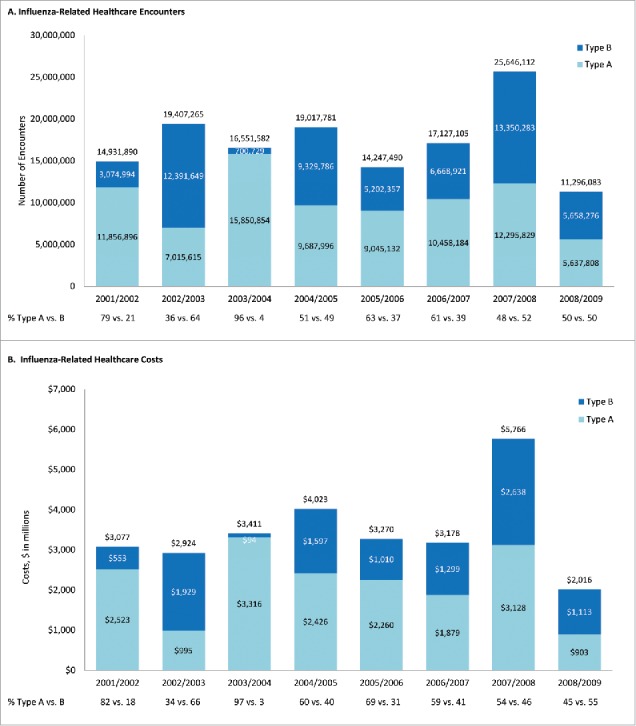
Projected influenza-related healthcare encounters and costs during 2001/2002 – 2008/2009, by influenza season and strain.

**Figure 2.**
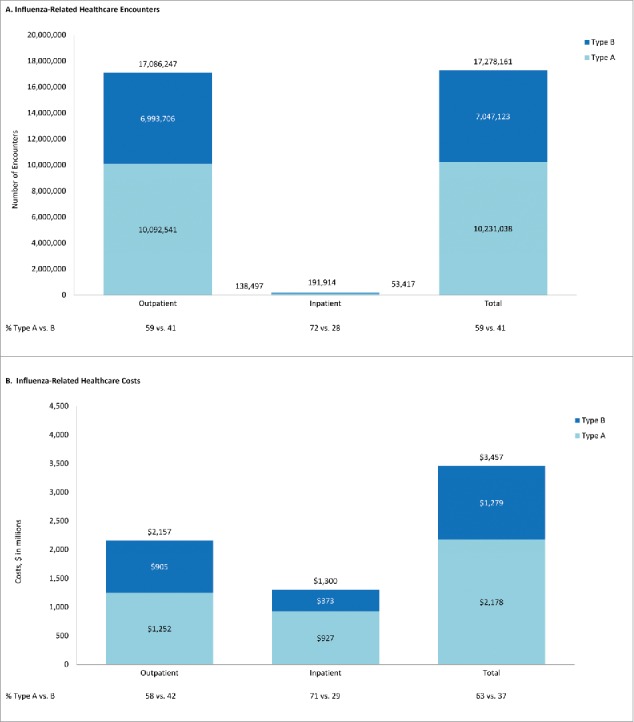
Projected influenza-related healthcare encounters and costs during 2001/2002 – 2008/2009, by care setting and strain.

During the period of interest, the projected annual number of influenza-related healthcare encounters in the US ranged from 11.3 million (2008/2009) to 25.6 million (2007/2008) (Fig. 1A); the mean number of healthcare encounters across all seasons was 17.3 million (Fig. 2A). Total annual healthcare costs ranged from $2.0 billion (2008/2009) to $5.8 billion (2007/2008) (Fig. 1B); across all seasons, average influenza cost was $3.5 billion (Fig. 2B). Annual levels of burden attributed to type A and B illness varied substantially across influenza seasons, with type B illness accounting for only 3% (2003/2004) to as much as 66% (2002/2003) of influenza-related healthcare costs; type-specific proportions were similar for influenza-related healthcare encounters (Fig. 1). At the person level, and across all seasons, projected total costs were $11.64 per year; 63% ($7.35) attributable to viral strain A, and 37% ($4.29) to viral strain B.

Influenza care in outpatient settings accounted for the majority of healthcare encounters when averaged over all seasons. Among the 17.1 million estimated influenza-related outpatient visits, 59% were attributed to type A illness and 41% to type B illness (Fig. 2A). Projected costs for outpatient encounters, including pharmacotherapy, were $2.2 billion overall; 58% for type A illness and 42% for type B illness (Fig. 2B). Pharmacotherapy accounted for <5% of outpatient costs. ED visits accounted for 6% of outpatient influenza-related encounters, for a projected average yearly cost of $494 million (Table 1). Projected annual cost of influenza-related hospitalization was $1.3 billion; type A illness accounted for 71%, and type B illness accounted for 29% of estimated hospital costs (Table 1).

**Table 1.** Projected influenza-related healthcare encounters and costs, by age, care setting, and strain (%A vs %B).

	Encounters and Costs, by Age (years)						
	0 – 4	5 – 17	18 – 49	50 – 64	65 – 74	≥ 75	All Ages
Encounters, No. (A% vs B%)							
Outpatient	2,003,195	5,968,322	6,421,302	1,635,901	557,045	500,482	17,086,247
	(75 vs 25)	(62 vs 38)	(53 vs 47)	(43 vs 57)	(77 vs 23)	(77 vs 23)	(59 vs 41)
* Physician Office*	*1,782,413*	*5,524,291*	*5,671,391*	*1,492,225*	*508,712*	*447,614*	15,426,646
* Emergency Dept.*	*197,683*	*294,871*	*479,992*	*71,672*	*24,905*	*32,791*	1,101,914
* Hospital Outpatient*	*23,099*	*149,160*	*269,919*	*72,004*	*23,428*	*20,077*	557,687
Inpatient	18,495	4,785	29,156	38,453	32,008	69,017	191,914
	(55 vs 45)	(61 vs 39)	(70 vs 30)	(73 vs 27)	(80 vs 20)	(74 vs 26)	(72 vs 28)
Total	2,021,690	5,973,107	6,450,458	1,674,354	589,053	569,499	17,278,161
	(75 vs 25)	(62 vs 38)	(53 vs 47)	(44 vs 56)	(77 vs 23)	(76 vs 24)	(59 vs 41)
Costs, $ in millions (A% vs B%)							
Outpatient^*^	$251	$655	$914	$212	$66	$59	$2,157
	(75 vs 25)	(61 vs 39)	(52 vs 48)	(42 vs 58)	(76 vs 24)	(77 vs 23)	(58 vs 42)
* Physician Office*	$161	$453	$502	$134	$47	$38	$1,335
* Emergency Dept.*	$86	$121	$224	$36	$11	$16	$494
* Hospital Outpatient*	$4	$81	$188	$42	$8	$5	$328
Inpatient	$65	$22	$194	$307	$252	$460	$1,300
	(54 vs 46)	(59 vs 41)	(70 vs 30)	(72 vs 28)	(79 vs 21)	(70 vs 30)	(71 vs 29)
Total	$316	$677	$1,108	$519	$318	$519	$3,457
	(71 vs 29)	(61 vs 39)	(55 vs 45)	(60 vs 40)	(79 vs 21)	(71 vs 29)	(63 vs 37)

* Outpatient costs include influenza-related pharmacotherapy

Findings for specific age groups are presented in Table 1. Approximately 28% of all hospitalizations were attributable to type B influenza; this percentage was much higher for type B illness in the 0–4 year-old group (45%) and the 5–17 year-old group (39%). In the 50–64 year-old group, total estimate of outpatient encounters attributed to type B illness (57%) exceeded estimates for type A illness (43%). Age-specific percentages of overall healthcare costs attributable to type B influenza were: 0–4 years, 29%; 5–17 years, 39%; 18–49 years, 45%; 50–64 year, 40%; 65–74 years, 21%; and ≥ 75 years, 29%.

### Sensitivity analyses

In one-way deterministic sensitivity analyses (Table 2), estimated total annual influenza-related healthcare costs were insensitive to changes in rates and unit costs of influenza-related rates, when such changes were based on corresponding 95% CIs from source materials. Results were somewhat more sensitive to changes in parameters for type A influenza vs. type B influenza. When evaluating parameters of uncertainty in the 1,000 replications of the PSA, estimates of total healthcare costs varied from $3.45–3.46 billion (mean, $3.45 billion).

**Table 2.** One-way deterministic sensitivity analyses on total annual costs of type A and type B influenza.

	Total Annual Cost of Influenza-Illness (millions)
Basecase	$3,458
Rates	
Type A	
Hospitalizations	
Lower Bound = −3%	$3,428
Upper Bound = 3%	$3,488
Physician Office Visits	
Lower Bound = −21%	$3,291
Upper Bound = 21%	$3,625
Emergency Department Visits	
Lower Bound = −21%	$3,395
Upper Bound = 21%	$3,520
Hospital Outpatient Visits	
Lower Bound = −21%	$3,420
Upper Bound = 21%	$3,496
Type B	
Hospitalizations	
Lower Bound = −3%	$3,448
Upper Bound = 3%	$3,468
Physician Office Visits	
Lower Bound = −17%	$3,362
Upper Bound = 17%	$3,554
Emergency Department Visits	
Lower Bound = −17%	$3,423
Upper Bound = 17%	$3,493
Hospital Outpatient Visits	
Lower Bound = −17%	$3,432
Upper Bound = 17%	$3,484
Unit Costs	
Type A	
Hospitalizations	
Lower Bound = −10%	$3,365
Upper Bound = 10%	$3,551
Physician Office Visits	
Lower Bound = −8%	$3,399
Upper Bound = 8%	$3,517
Emergency Department Visits	
Lower Bound = −22%	$3,393
Upper Bound = 22%	$3,523
Hospital Outpatient Visits	
Lower Bound = −32%	$3,402
Upper Bound = 32%	$3,514
Type B	
Hospitalizations	
Lower Bound = −10%	$3,421
Upper Bound = 10%	$3,495
Physician Office Visits	
Lower Bound = −8%	$3,416
Upper Bound = 8%	$3,500
Emergency Department Visits	
Lower Bound = −22%	$3,413
Upper Bound = 22%	$3,503
Hospital Outpatient Visits	
Lower Bound = −32%	$3,411
Upper Bound = 32%	$3,505

## Discussion

We developed a model to estimate US healthcare costs of illness attributable to type A and type B influenza from 2001 – 2009. Overall, influenza was associated with substantial clinical and economic burden, and type B influenza illness was responsible for a considerable percentage of this burden, accounting for an average of 41% of healthcare encounters and 37% of healthcare costs. In one of the 8 seasons evaluated, type B illness accounted for 66% of influenza-related healthcare costs. Although our focus was on healthcare costs, the disproportionately high impact of type B illness during highly productive periods of life, both in terms of paid employment and child rearing activities, is noteworthy and warrants further study. Type B illness among very young children, especially encounters requiring inpatient care and the corresponding high cost burden of hospitalization also is a noteworthy finding. While it was not possible for us to characterize results separately for children aged <1 y and 1–4 years, respectively, due to limitations of the source material, it is more than likely that influenza-related healthcare costs are not uniformly distributed among children aged <4 y and that the burden among children aged <1 y is disproportionately greater than it is among those aged 1–4 years.^7^

Taken collectively, these findings suggest that concerted efforts aimed at increasing influenza vaccine coverage — especially with the recently approved quadrivalent products,^9^ which include an additional type B strain — could yield substantial cost savings to the US healthcare system. Reduction of influenza burden could also help to alleviate access to care issues in emergency departments, where an important minority of patients receive influenza-related treatment. Additionally, many people at risk of influenza, especially older people, live with comorbidities such as diabetes, chronic lung conditions, cancer, and kidney or heart problems, and influenza is the 7^th^ leading cause of death among persons aged ≥ 65 y.^10,11^ Broader vaccine coverage has the potential to reduce destabilization of such pre-existing conditions for many persons, with resulting clinical and economic benefits. While influenza vaccination imparts direct health benefits to the person immunized, it also may confer indirect benefits (i.e., via herd effects) by decreasing disease transmission to others, and thus the positive impact of broader influenza vaccine (type B) coverage in decreasing overall influenza burden could be substantive.

While our primary focus was on elucidating the proportion of the annual burden of influenza attributable to type A vs. type B illness, we note our projections of influenza-related encounters and costs are lower than those reported by Molinari and colleagues.^5^ Using a probabilistic modeling approach and publically available epidemiologic data, Molinari *et al*. estimated that the annual burden of influenza included 31.4 million outpatient visits, 334,185 hospitalizations, and direct medical costs of $10.4 billion. Using a more conservative approach, their “lower bound” estimates were 128,710 hospitalizations and $5.8 billion in direct medical costs (the corresponding number of outpatient visits was not reported). Differences in both underlying rates of disease as well as (unit) costs of encounters largely explain these discrepancies. For example, Molinari *et al*. estimated the cost of hospitalization based on data from a large healthcare claims database and included—in the episode—all claims with pneumonia/influenza diagnoses during the period beginning 2 weeks before admission through 30 days post-discharge; estimated costs were based on paid (i.e., reimbursed amounts) and ranged from $10,880 for non-high-risk children aged <5 y to $267,954 for high-risk children aged <18 y. In our study, the cost of hospitalization was based on a hospital records database and thus was limited to the period of admission; reported charges were stepped down to estimates of costs (which are typically lower than reimbursed amounts), and ranged from $2,891 for children aged 0–4 years (2001/2002) to $10,085 for adults aged 50–74 years (2008/2009).

We note several limitations of our study. First, rates of influenza-related healthcare encounters were based on 2 retrospective studies that utilized secondary databases and ICD-9-CM diagnostic codes. The sensitivity and specificity of the operational algorithms used in these studies to identify the events of interest are unknown, and assessment of their accuracy is beyond the scope of this study. We note that methods for estimating health outcomes related to influenza were first published over a decade ago and have been used in a number of epidemiologic evaluations.^12^ Second, we used unit-costing methods based on data from one influenza season (2009) and expressed price levels prevailing in each year using the CPI. Such adjustment did not take into account changes in clinical practice over time, which could have influenced the composition and resulting costs of services provided. Third, when estimating the cost (unit) of influenza-related hospitalizations using the HCUP-NIS, attention was limited to admissions with a qualifying diagnosis in the primary position. It is possible that patients with other acute presentations or with chronic comorbidities received care unrelated to influenza while hospitalized. This has the potential to upwardly bias the magnitude of estimated hospital costs; however, there is no reason to believe this would differentially affect persons with types A or B influenza. Fourth, information on inpatient drug utilization is not available in the HCUP-NIS, and while it is theoretically possible to identify the use of other drugs (i.e., besides antivirals and antibiotics) that may be related to influenza care in the outpatient setting (i.e., using the MEPS-HC), determining the precise reason for use is not straightforward and including some of these other drugs has the potential to upwardly bias our estimates (e.g., if such drugs were used for non-influenza reasons). For this reason, the use of other drugs was not considered in our analysis. Fifth, resource utilization rates and unit costs were – as feasible – obtained from nationally representative sources, and to the extent rates and unit costs vary across subgroups defined on the basis of important factors (e.g., type of health insurance), caution should be exercised when generalizing study results to subgroups and other populations. Sixth, this study did not address the burden of influenza among persons who did not seek care, and did not consider morbidity- and mortality-related productivity loss. We also did not estimate the adverse impact on quality of life from influenza. Finally, we used a conservative estimate of influenza-related healthcare encounters employed by Matias and colleagues, focusing on respiratory-related events without considering cardiac events that may be associated with influenza.

In summary, the results of this study suggest that influenza was associated with considerable clinical and economic burden in each year during the periods of interest, and that, while the proportion of viral type A illness was larger in most areas of our analyses, the burden of influenza-related illness attributable to type B influenza virus was substantial. Increasing vaccine coverage, especially with the recently developed quadrivalent products, has the potential to reduce the overall annual burden of influenza in the US.^13^ The findings of this investigation may serve as a benchmark against which the benefits from broader use of quadrivalent vaccines for the US population may be evaluated.

## Methods

### Model overview

We developed a model using historic projections of type-specific influenza rates to estimate annual healthcare encounters and costs of influenza in the US over 8 seasons. Healthcare costs were estimated for influenza type A and influenza type B illness separately and collectively by influenza season and age group based on:

· Seasonal rates of influenza-related illness requiring formal healthcare from 2001/2002 – 2008/2009;
· Unit costs of influenza-related healthcare encounters;
· Estimates of population size for the US in each influenza season of interest.

Influenza-related encounters requiring formal healthcare included both hospitalizations and outpatient visits. The latter was further stratified by setting of care — physician office, emergency department (ED), and hospital outpatient settings. Total direct healthcare cost of influenza-attributable illness was estimated across all care settings (i.e., including the cost of inpatient and outpatient care) and by specific healthcare settings (i.e., for inpatient and outpatient care separately) during each influenza season. Healthcare cost was tallied on an overall basis (i.e., collectively for type A and type B illness) and by influenza type (i.e., separately for type A and type B illness), on an age-specific basis and as well as for all persons. The cost of influenza vaccination was not considered as our focus was on the burden associated with influenza illness, and not the cost of prevention. A detailed listing of all model inputs is contained in the **online supplement: Model input parameter values**.

### Model estimation

#### Model population

Estimates of the size of the US population, overall and by age groups, were derived using national census-based information for calendar years 2001–2009.^14^ Yearly estimates spanned the period from July 1^st^ to June 30^st^. Estimates corresponding to population size at the end of each influenza season were used in the model (e.g., for flu season 2001/2002, the population size reported for July 2002 was used).

#### Rates of encounters

Rates of influenza-related hospitalizations during the respective seasons were obtained from a recently completed evaluation of the clinical burden of severe influenza in the US from July 1997 to April 2009.^15^ In this study by Matias and colleagues, rates of influenza-attributable hospitalizations were estimated on an overall basis as well as by age, calendar year, influenza type (A and B), and influenza subtype (A/H3N2 and A/H1N1) based on data from the Healthcare Cost and Utilization Project Nationwide Inpatient Sample (HCUP-NIS) and from FluView, the weekly influenza update by the US CDC. Influenza-attributable hospitalizations included all those coded to the category “all diseases of the respiratory system plus cough, abnormalities of breathing, fever, and viral infections not otherwise specified (NOS),” and were identified in the HCUP-NIS based on principal diagnoses (ICD-9-CM codes 460–519, 079, 786.0, 786.1–786.4, 786.7–786.9).

Rates of influenza-related outpatient visits were derived in large part from a similar evaluation by Matias and colleagues characterizing the clinical burden of mild influenza in the US from July 2000 to June 2011.^16^ In this study, Matias and colleagues reported estimated rates of influenza-attributable physician office visits on an overall basis as well as by age, calendar year, influenza type, and influenza subtype based on data from a large US private healthcare claims database. Because Matias *et al.* considered only physician office visits, rates of ambulatory encounters in other settings (i.e., emergency department [ED] and hospital outpatient) were derived using the Matias data and the 2009 Household Component of the Medical Expenditure Panel Survey (MEPS-HC).^17^ Specifically, rate ratios (and 95% Confidence Intervals (CIs)) for these other ambulatory settings relative to the physician office were estimated using MEPS-HC data, and rate ratios for ED and hospital outpatient were then combined with rates of influenza-related physician office visits from the Matias study to derive rates of influenza-related ED and hospital outpatient visits during 2001/2002 – 2008/2009. In these analyses, all outpatient encounters for respiratory-related reasons first were identified using the ICD-9-CM codes set forth above, and all such encounters were then categorized by corresponding type/place of service.

#### Costs of encounters

Cost of influenza-related hospitalizations included those for acute-care facility services and associated physician visits (including admission, daily, and discharge visits). The former was estimated using mean age-specific hospital charges for all admissions with a principal diagnosis of respiratory-related disorders, consistent with methods employed in the studies by Matias and colleagues, in the 2009 version of the HCUP-NIS.^18^ Hospital charges were stepped down to estimates of hospital cost by multiplying the former by facility-specific cost-to-charge ratios that are provided in the HCUP-NIS database. The number and cost of physician visits were estimated using age-specific estimates of length of stay (LOS) in hospital for respiratory-related admissions from the 2009 version of the HCUP-NIS and published physician fee schedules, respectively.^19^ Hospitalizations were flagged only if they occurred during the influenza season, which was defined based on information from the US CDC (CDC 2013). Estimated hospital costs were expressed at price levels prevailing in each of the years of interest (i.e., 2001/2009 – 2008/2009); the inpatient hospital service component of the US Consumer Price Index (CPI) for All Urban Consumers was used to deflate cost estimates from 2009 to earlier study years.^20^

The cost of outpatient visits for influenza illness was estimated based on analyses of data from the 2009 MEPS-HC. Mean payments (including expenditures by payers and patients) for visits with a diagnosis in any position of respiratory-related disorders, defined above, were included. Only outpatient visits that occurred during the influenza season, based on information from the US CDC (CDC 2013), were considered in estimating unit costs. The cost of influenza-related outpatient visits was estimated separately for those occurring in a physician office, emergency department, and hospital outpatient setting. These encounters and costs were further evaluated by age. Estimated costs were expressed at price levels prevailing in each of the years of interest; the medical care component of the US CPI for All Urban Consumers was used to deflate (unit) cost estimates from 2009 to earlier study years.^20^ Pharmacotherapy use and cost were estimated for outpatient visits based on data from the 2009 MEPS-HC, and included selected antiviral and antibiotic agents. A complete listing of all model input values is set forth in the online supplement.

### Analyses

The numbers and direct costs of healthcare encounters in the US during each of 8 influenza seasons from 2001/2002 – 2008/2009 were estimated. Outcomes and costs were reported on an overall basis and by type A and type B illness, for each season and age group. To evaluate the stability of our findings, we conducted one-way deterministic sensitivity analyses (DSA); upper and lower bounds of input parameters were based on 95% confidence intervals (CIs). Probabilistic sensitivity analyses (PSA) were undertaken to generate 95% CIs for total healthcare costs attributable to type A and type B illness, accounting for uncertainty surrounding rates and unit costs. A total of 1,000 replications were performed. Standard errors were estimated using data from the source materials as well as a beta distribution for rates and a normal distribution for costs.

## Supplementary Material

2017HV0153R-s01.xlsx

## Abbreviations

CI: confidence interval
CPI: consumer price index
DSA: deterministic sensitivity analyses
ED: emergency department
LOS: length of stay
MEPS-HC: Household Component of the Medical Expenditure Panel Survey
NOS: not otherwise specified
PSA: probabilistic sensitivity analyses
US: United States
